# BRCA2 gene mutation in cancer

**DOI:** 10.1097/MD.0000000000031705

**Published:** 2022-11-11

**Authors:** Chunbao Xie, Jiangrong Luo, Yangjun He, Lingxi Jiang, Ling Zhong, Yi Shi

**Affiliations:** a Department of Laboratory Medicine, Sichuan Provincial People’s Hospital, University of Electronic Science and Technology of China, Chengdu, China; b Department of Anesthesiology, Sichuan Academy of Medical Sciences & Sichuan Provincial People’s Hospital, Chengdu, China; c Department of Medical Laboratory, Southwest Medical University, Luzhou, China; d Health Management Center, Sichuan Provincial People’s Hospital, University of Electronic Science and Technology of China, Chengdu, China; e Sichuan Provincial Key Laboratory for Human Disease Gene Study and Department of Laboratory Medicine, Sichuan Provincial People’s Hospital, University of Electronic Science and Technology of China, Chengdu, China; f Research Unit for Blindness Prevention of Chinese Academy of Medical Sciences (2019RU026), Sichuan Academy of Medical Sciences & Sichuan Provincial People’s Hospital, Chengdu, Sichuan, China.

**Keywords:** BRCA2, breast cancer, ovarian cancer, PRAP inhibitor, prostate cancer

## Abstract

Breast cancer susceptibility gene 2 (BRCA2) is the main gene associated with hereditary breast cancers. However, a mutation in BRCA2 has also been found in other tumors, such as ovarian, pancreatic, thyroid, gastric, laryngeal, and prostate cancers. In this review, we discuss the biological functions of BRCA2 and the role of BRCA2 mutations in tumor progression and therapy.

## 1. Introduction

Breast cancer susceptibility gene 2 (BRCA2), which was discovered in 1995, is expressed in various tissues as part of the normal genetic architecture inherent to all humans. The most common mutations in *BRCA2* are shift and missense mutations, with exon 11 being the most common mutation site.^[1]^ Germline mutations occur, they may lead to hereditary breast and ovarian cancer syndrome, which accounts for 5% to 7% of all breast cancer cases. Patients with hereditary breast and ovarian cancer syndrome have a 50% to 80% risk of developing breast cancer and 30% to 50% risk of developing ovarian cancer. This syndrome is not only associated with early onset breast and ovarian cancer but also increases the risk of pancreatic, gastric, laryngeal, fallopian tube, malignant skin, eye melanoma, and prostate cancer.^[2,3]^ In this review, we discuss the biological functions of *BRCA2* and the role of BRCA2 mutations in tumor progression and therapy.

## 2. *BRCA2* gene

### 2.1. BRCA2 gene structure and function

**Figure 1.**
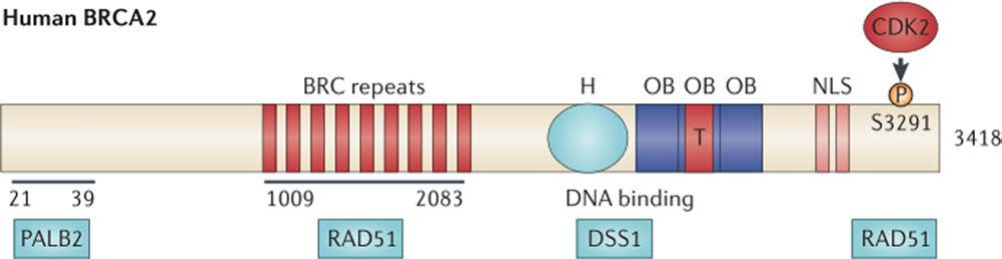
Basic structure and binding protein of the BRCA2 gene. BRCA2 = breast cancer susceptibility gene 2.

The *BRCA2* is located in region 12 of the long arm of chromosome 13 and consists of 27 coding exons, the largest being exon 11 (4.9 kb). *BRCA2* encodes a protein of 3418 amino acids (10.2 kb). The N-terminus of *BRCA2* binds to PALB2. In addition, BRCA2 contains 8 BRC repeats located between amino acid residues 1009 to 2083 and binds to RAD51. The *BRCA2* gene DNA binding domain contains 5 components: a 190 amino acid alpha helix domain (H), 3 oligonucleotide binding folds (single-stranded DNA binding module), and a tower-like domain (T) that extends from 3 oligonucleotide binding 2 and binds to dsDNA to bind single-stranded DNA. The C-terminus of *BRCA2* contains an NLS and a phosphorylation site S3291 for cyclin-dependent kinase, which also binds to RAD51 (Fig. 1).^[4]^

The *BRCA2* gene mediates the recruitment of RAD51 filaments to the DNA double-strand break site and interacts in the repair process to form the RAD51-BRCA2-DSS1 complex, which uses sister chromatids as a template to repair damage through “error-free” homologous recombination.^[5]^ The *BRCA2* gene also appears to participate in cytoplasmic division; when its function is disrupted, cytoplasmic division is impaired, and the incidence of binucleated cells is increased.^[6]^ Studies have also shown that *BRCA2* gene-deficient cells exhibit alterations in chromosome number (aneuploidy) as well as structurally abnormal chromosomes, resulting in *BRCA2* gene-deficient tumors that are frequently aneuploid.^[7]^ In addition to its DNA repair function, *BRCA2* inhibits tumor development by suppressing cancer cell growth.^[8]^

### 2.2. Mutations in the BRCA2 gene

There are 2 types of BRCA mutations (BRCAm): germline mutations (gBRCAm) (also known as germline mutations), which can be passed on to the offspring; and somatic mutations (sBRCAm), which are non-inherited mutations. The former refers to mutations in sperm or egg cells that cause all somatic cells of an individual to carry the mutated gene, and are inherited through autosomal dominant transmission to the offspring. sBRCAm refers to mutations that occur in cancerous somatic cells and are not passed on to offspring. In clinical studies, BRCAm is referred to as gBRCAm. Pathogenic mutations in *BRCA2* include shift mutations, missense mutations, nonsense mutations, and deletion mutations, mostly shift and nonsense mutations.^[1]^ When mutations occur, the DNA double-strand break repair process does not proceed normally and the upstream codon encoding the amino acid is converted to a stop codon, which in turn terminates transcription and leads to the formation of short-cut proteins that affect protein function. Their loss of function easily leads to genetic instability, leading to a varying degree of increased cancer risk in BRCA2 mutation carriers compared with the general population.^[9]^

BRCA2 mutant cells have recombination defects, and patients with BRCA2 mutations are more sensitive to chemotherapy than patients with breast cancer susceptibility gene 1 (BRCA1) mutations. Studies have also shown a difference between BRCA1 and BRCA2 mutations and patient survival in epithelial ovarian cancer, with patients with BRCA2 mutations having a better prognosis than those with BRCA1 mutations.^[10]^ In addition to pathogenic mutations in the *BRCA2*, there are also multi-base locus mutations and single nucleotide mutations^[11]^; however, the relationship between such mutations and ovarian cancer is still not fully understood.

### 2.3. BRCA2 gene mutation loci

To date, 3 founder mutations (BRCA1 185delA, BRCA1 5382 insC, and BRCA2 6174delT) have been extensively studied owing to their relatively high incidence. BRCA1 1100delAT and BRCA1 5589del8 were found to have a partial ancestral effect in the Han Chinese population^[12]^ and may be used as biomarkers to assess the risk of developing breast, ovarian, and other cancers in women. Another study showed that the Korean population had the highest rate of c.7480C>T mutations in *BRCA2*,^[13]^ while c.3109C>T mutations were more common in the Chinese.^[14]^ Moreover, a study from eastern Spain found that *BRCA2* gene mutations with high recurrence rates were c.9026_9030delATCAT, c.3264 insT, and c.8978_8991del14, accounting for 43.2% of all mutations in this gene, and c.9026_9030delATCAT was the most common mutation, accounting for 21.3% of BRCA2 gene mutations.^[15]^ In addition, a small-sample survey in China found that the frequency of missense mutations at the c.7397T > c locus was the highest among several *BRCA2* gene mutation loci.^[16]^ With the development of genetic testing technology, this class of genes can be tested and the risk of developing tumors can be further assessed.

### 2.4. BRCA2 mutations and PARP inhibitors

Poly ADP-ribose polymerase (PARP) has a key role in the DNA repair pathway. PARP binds to DNA gaps and ends. Seventeen members of the PARP family have homologous catalytic regions.

There are 2 main types of DNA damage: single-strand breaks (SSBs); and double-strand breaks. The most common type of DNA damage is SSB, which arises following base modification or deletion. PARP-1 senses SSB through its DNA-binding domain and undergoes poly ADP PARylation (ribosylation modification), activating PARP-1, which then binds to XRCC1 (a scaffolding protein for other DNA repair enzymes). In addition, other DNA repair enzymes, such as DNA ligase-3, DNA polymerase beta, and bifunctional polynucleotide kinase 30 phosphatase (PNKP) enter the site of DNA damage and bind to XRCC1 to form the SSB repair complex (SSBRC), which ultimately repairs the damaged DNA.

DNA double-strand breaks are mainly caused by ionizing radiation or when DNA replication is interrupted. Cells use homologous recombination and non-homologous end-joining mechanisms to repair double-strand breaks in DNA.^[17]^

PARP inhibitors are mainly composed of NAD+ analogs that compete with NAD+ to bind to the active site of the catalytic structural domain of PARP, causing inhibition of PARP activity, which in turn affects the formation of PARP1-ADP ribose branched chains and prevents them from recruiting DNA damage-associated repair proteins, ultimately resulting in failure of DNA damage repair.^[18]^

Mechanism of action in synthetic lethality of recombinant DNA repair (HRR)-deficient cells: Endogenously induced DNA single-strand damage is normally repaired by PARP-dependent single-strand damage repair, leading to cell survival. If PARP is inhibited, single-strand DNA damage accumulates, leading to replication fork collapse. Replication fork collapse is repaired by cellular DNA homologous recombination repair, where BRCA1, BRCA2, and several other proteins are involved in the repair process, preparing DNA ends, loading RAD51 onto single-stranded DNA, and subsequently allowing the homologous strand to invade the complementary double strand and serve as a template for DNA synthesis and high-fidelity DNA repair. In cases where homologous repair is disrupted (e.g., in BRCA mutant cells), replication fork collapse cannot be repaired, and the cell dies.^[19]^

PARP inhibitors selectively kill tumor cells with defective homologous recombination function caused by BRCA gene mutations, but have no effect on cells with normal *BRCA* genes.^[20]^ The efficacy of PARP inhibitors has been demonstrated in patients with advanced ovarian cancer and BRCA1/2 mutations. Moreover, maintenance treatment with Olaparib provided substantial progression-free survival, with a 70% reduction in the risk of disease progression or death compared with placebo.^[21]^

## 3. The BRCA2 gene and cancer

### 3.1. *Breast cancer*

In 2020, female breast cancer has become the most common cancer globally, with an estimated 2.3 million new cases (11.7%) and 6.9% of all cancer-related deaths in women. Breast cancer accounts for 1-quarter of all cancer cases and 1 to 6th of all cancer-related deaths among women. In most countries, breast cancer is the leading cause of morbidity and mortality.^[22]^ A recent survey suggested that the annual incidence of breast cancer in China is 420,000 cases.^[22]^

Approximately 10% of breast cancer cases are caused by pathogenic germline mutations in identified breast cancer susceptibility genes, also known as hereditary breast cancer.^[23]^ The incidence of breast cancer in *BRCA2* mutation carriers is 12.50%, which is 5 times that in non-mutation carriers (2.49%).^[24]^ The cumulative risk of breast cancer in healthy European and American women aged 80 years who carry the *BRCA2* mutation is 69%, and the risk of breast cancer in healthy carriers with 2 or more breast cancer cases in their family lineage is approximately twice that of carriers without a family history.^[25]^ A Chinese study showed that the cumulative risk of breast cancer in healthy Chinese women aged 70 years who carried a *BRCA2* mutation was 36.5%, which was approximately 10 times the risk of breast cancer in an average healthy Chinese woman (3.6%).^[26]^ In addition, carriers of germline mutations in the *BRCA1* and *BRCA2* genes have an average age of onset 7 years earlier than carriers without mutations, and those carrying both *BRCA1* and *BRCA2* gene mutations have an earlier age of onset than those with a single mutation.^[27]^ Moreover, breast cancer-specific survival is worse in carriers of *BRCA2* mutations than in sporadic cases.^[28]^ Moreover, BRCA2 is expressed at a higher level in normal breast tissue (90% expression) than in invasive ductal carcinoma of the breast (69.1% expression).^[29]^

Early diagnosis of single nucleotide polymorphism loci in the susceptibility gene *BRCA2* not only allows early prevention, diagnosis, and treatment of patients with BRCA2-associated breast cancer but may also help identify new therapeutic targets at the genetic level, providing a scientific basis for individualized prevention and treatment of breast cancer and further guiding the diagnosis and treatment.^[30]^

Mastectomy is effective in preventing the development of breast cancer. Prospective studies have shown that mastectomy reduces the risk of breast cancer by 90% or more, with a residual risk of 1% to 2% in BRCA1 and BRCA2 mutation carriers. Without risk-reducing mastectomy, the risk of breast cancer in BRCA1 and BRCA2 mutation carriers is approximately 70%.^[25]^ The NCCN recommends risk-reducing tubo-oophorectomy for women with the *BRCA2* mutation (aged 40‐45 years), as it can significantly reduce the incidence of ovarian cancer.^[31]^

Regarding pharmacological prevention, only a small sample of retrospective studies has shown that tamoxifen reduces the risk of breast cancer in healthy carriers of the *BRCA2* gene mutation by 62% (288 cases, 11 with BRCA2 mutation).^[32]^

### 3.2. Ovarian cancer

Ovarian cancer is a common cause of death in gynecological cancers. Due to the lack of obvious clinical symptoms in the early stages and the lack of effective screening methods, approximately 70% of patients with epithelial ovarian cancer present with advanced stage disease at the time of diagnosis.^[33]^ Approximately 20 genes are associated with ovarian cancer, with *BRCA* having the most significant impact. Mutations in the *BRCA* gene are responsible for approximately 80% of genetically associated ovarian cancers.^[34]^ The association between BRCA mutations and ovarian cancer mortality has been reported in many studies; however, the results remain debatable. Some researchers have found that ovarian cancer patients with BRCA mutations have a better prognosis, whereas others have shown null results. A previous study found that mutations in *BRCA2* increase the risk of ovarian cancer by approximately 11.4%.^[35]^

The most common treatments include surgery and chemotherapy with platinum-containing regimens. For epithelial ovarian cancer (BMOC) with *BRCA* gene mutations, platinum-based therapies are the treatment of choice for EOC of epithelial ovarian cancer, and BMOC patients are more sensitive to platinum-based chemotherapy than patients with wild-type *BRCA* genes (i.e., no mutation in the *BRCA* gene).^[36]^

### 3.3. Prostate cancer

Prostate cancer is the most common cancer in men. Studies have suggested that prostate cancer patients account for 26% of diagnosed cancer cases in men,^[37]^ with approximately 900,000 new cases each year. The prevalence of prostate cancer varies between regions, with Australia, New Zealand, North America, Western Europe, and Northern European countries having the highest incidence of prostate cancer and South Central Asian countries having the lowest incidence. The genetic etiology of prostate cancer is complex and poorly studied, with multiple factors contributing to its development.^[38]^

*BRCA2* is highly expressed in prostate cancer tissues, and silencing of this gene can block the activation of the ATM signaling pathway, thereby inhibiting the proliferation of prostate cancer cells and promoting their apoptosis.^[39]^ In addition, it has been suggested that BRCA mutations are not associated with prostate cancer development. Studies have demonstrated that BRCA2 mutations increase the risk of prostate cancer by 8.3-fold. Also, Mutations in the *BRCA2* gene have been associated with an increased risk of high-grade disease, progression of metastatic denervation-resistant disease, and a 50 to 60% cancer-specific 5-year survival rate.^[40]^ Patients with metastatic destructive-resistant prostate cancer with germline BRCA mutations (versus no mutations) seem to respond well to first-line neoadjuvant hormonal ablative therapy (NHT).^[41]^ In addition, the PARP inhibitor olaparib is effective in BRCA-associated cancers, especially prostate cancer.^[42]^

### 3.4. Thyroid cancer

Thyroid cancer is the most common malignant endocrine disease. Studies have shown a rapid increase in the incidence of thyroid cancer over the past few decades, which has resulted in widespread public concern.^[43]^ Recent studies have suggested that decreased BRCA2 expression may be associated with the development and progression of sporadic thyroid cancer.^[44,45]^

### 3.5. Lung cancer

BRCA2 was downregulated in all 6 lung squamous carcinoma specimens. Moreover, *BRCA2* has been suggested as a molecular marker for lung squamous carcinoma, providing a direction for further study of lung squamous carcinoma.^[46]^

### 3.6. Pancreatic cancer

Studies have shown that the incidence of pancreatic cancer has been increasing annually over the past few decades, and this increasing trend has caused widespread public concern.^[47]^ Pancreatic cancer is a highly malignant tumor of the gastrointestinal tract. According to the latest global cancer burden report, pancreatic cancer ranks 7^th^ in global cancer-related mortality rate and 8^th^ in the number of new cancer cases in China. To date, only effective therapies have been developed to treat advanced diseases.

Germline *BRCA* mutations significantly increase the risk of pancreatic cancer. BRCA mutations have been found in up to 8% of the patients with sporadic pancreatic cancer. Platinum-based chemotherapy and poly (adp-ribose) polymerase inhibitors are effective treatment options for patients with germline BRCA mutations.^[48]^

The vast majority of pancreatic cancers are ductal adenocarcinomas of the exocrine pancreas (PDAC), and the majority of PDAC cases are considered disseminated. However, 5 to 10% of patients with a family history of PDAC are estimated to be familial, and there are at least 12 known genetic syndromes or genes associated with an increased risk of pancreatic cancer, the most prominent of which contains the *BRCA2* gene.^[49]^ The frequency of *BRCA* gene mutations, particularly in BRCA2, is also increasing in familial pancreatic cancer. In the case of *BRCA2* gene mutations, studies have found germline mutations in 3.7% to 19% of patients with a strong family history of PDAC. Moreover, a study investigating 180 American families identified 10 carriers, suggesting that BRCA2 mutations accounted for 6% of families with moderate- and high-risk pancreatic cancer.^[50]^ The prevalence of deleterious mutations in the *BRCA2* (excluding variants of unknown significance) was 3.7% among family members with prior evidence of pancreatic cancer. An earlier study by the Breast Cancer Chain Association found a 3.5-fold increased risk of pancreatic cancer in *BRCA2* mutations.^[51]^

## 4. Summary and outlook

BRCA2 mutation carriers of cancer have different clinicopathological features, chemotherapeutic drug sensitivity, and targeted therapy in cancer patients with no mutations. In addition, different treatment approaches, including surgery, chemotherapy regimens, and targeted therapy, may be used for these patients. Currently, BRCA2 mutated tumors are more sensitive to platinum-based agents and PARP inhibitors. However, there is an urgent need to address the issue of increased resistance to these agents. In addition, accurate risk assessment of healthy female carriers in the family of these patients should be carried out as far as possible, and appropriate preventive measures should be developed based on the level of risk, which is essential to reduce the incidence in healthy carriers.

## Author contributions

**Conceptualization:** Yi Shi.

**Formal analysis:** Yangjun He.

**Supervision:** Jiangrong Luo, Lingxi Jiang.

**Writing – original draft:** Chunbao Xie.

**Writing – review & editing:** Ling Zhong.
